# Gallium-68 Ventilation/Perfusion PET-CT and CT Pulmonary Angiography for Pulmonary Embolism Diagnosis: An Interobserver Agreement Study

**DOI:** 10.3389/fmed.2020.599901

**Published:** 2021-02-16

**Authors:** Romain Le Pennec, Amir Iravani, Beverley Woon, Brieg Dissaux, Bibiche Gest, Pierre-Yves Le Floch, Pierre-Yves Salaün, Grégoire Le Gal, Michael S. Hofman, Rodney J. Hicks, Pierre-Yves Le Roux

**Affiliations:** ^1^Nuclear Medicine, Brest University Hospital, EA3878 (GETBO) IFR 148, Brest, France; ^2^Molecular Imaging and Therapeutic Nuclear Medicine, Peter MacCallum Cancer Centre, Melbourne, VIC, Australia; ^3^Radiology, Brest University Hospital, EA3878 (GETBO) IFR 148, Brest, France; ^4^Department of Medicine, Ottawa Hospital Research Institute, University of Ottawa, Ottawa, ON, Canada; ^5^Sir Peter MacCallum Department of Oncology, University of Melbourne, Melbourne, VIC, Australia

**Keywords:** V/Q PET/CT, V/Q pulmonary imaging, interobserver agreement, interobservator variability, CTPA (computed tomography pulmonary angiography)

## Abstract

**Objectives:** ^68^Ga Ventilation/Perfusion V/Q PET-CT is a promising imaging tool for pulmonary embolism diagnosis. However, no study has verified whether the interpretation is reproducible between different observers. The aim of this study was to assess the interobserver agreement in the interpretation of V/Q PET-CT for the diagnosis of acute PE, and to compare it to the interobserver agreement of CTPA interpretation.

**Methods:** Twenty-four cancer patients with suspected acute PE underwent V/Q PET-CT and CTPA within 24 h as part of a prospective pilot study evaluating V/Q PET-CT for the management of patients with suspected PE. V/Q PET-CT and CTPA scans were reassessed independently by four nuclear medicine physicians and four radiologists, respectively. Physicians had different levels of expertise in reading V/Q scintigraphy and CTPA. Interpretation was blinded to the initial interpretation and any clinical information or imaging test result. For each modality, results were reported on a binary fashion. V/Q PET/CT scans were read as positive if there was at least one segmental or two subsegmental mismatched perfusion defects. CTPA scans were interpreted as positive if there was a constant intraluminal filling defect. Interobserver agreement was assessed by calculating kappa (κ) coefficients.

**Results:** Out of the 24 V/Q PET-CT scans, the diagnostic conclusion was concordantly negative in 22 patients and concordantly positive in one patient. The remaining scan was interpreted as positive by one reader and negative by three readers. Out of the 24 CTPA scans, the diagnostic conclusion was concordantly negative in 16 and concordantly positive in one. Out of the seven remaining scans, PE was reported by one reader in four cases, by two readers in two cases, by three readers in one case. Most of discordant results on CTPA were related to clots reported on subsegmental arteries. Mean kappa coefficient was 0.79 for V/Q PET-CT interpretation and 0.39 for CTPA interpretation.

**Conclusions:** Interobserver agreement in the interpretation of V/Q PET-CT for PE diagnosis was substantial (kappa 0.79) in a population with a low prevalence of significant PE. Agreement was lower with CTPA, mainly as a result of discrepancies at the level of the subsegmental arteries.

## Introduction

Pulmonary embolism (PE) is a common and potentially lethal condition but is also treatable (1). Untreated PE is reported to have a mortality rate of up to 30% (2). On the other hand, anticoagulant therapy is effective, but it is also expensive and is associated with the risk of bleeding (3). Therefore, an accurate diagnosis is required for all patients with suspected PE. Computed tomography pulmonary angiography (CTPA) and ventilation-perfusion (V/Q) lung scan are the two non-invasive procedures validated for the diagnosis of PE. However, both tests have some limitations. Limitations of CTPA include a potential higher radiation dose, the use of iodinated contrast media, and a possible overdiagnosis and overtreatment of PE (4, 5). On the other hand, planar V/Q scan leads to a higher proportion of non-diagnostic tests, although the introduction of single photon emission computed tomography (SPECT) and SPECT-CT has been reported to improve the diagnostic performance of V/Q imaging (6–8), although the CT component requires a SPECT/CT system and exposes to a slightly higher radiation dose.

V/Q PET-CT, positron emission tomography, is a new and promising imaging modality for PE diagnosis. The same carrier molecules as conventional V/Q scan are used (i.e., carbon nanoparticles for ventilation and macro-aggregated albumin for perfusion), but they are labeled with ^68^Ga instead of ^99m^Tc (9–11). V/Q PET-CT and V/Q SPECT-CT imaging evaluate similar physiological processes, but with the technical advantages of PET over conventional SPECT imaging, including higher sensitivity, greater spatial and temporal resolution, and more rapid scan acquisition (12–14). V/Q PET-CT showed promising results to define regional lung function (15, 16), with a view to optimizing radiotherapy planning or lung surgery in patients with lung cancer (17–19). Recently, our groups showed the feasibility and potential utility of V/Q PET-CT as compared with CTPA for the management of cancer patients with suspected acute PE (20). However, and so far, no study has verified whether the interpretation is reproducible between different physicians. Assessing the interobserver reliability of interpretation across readers, including more inexperienced clinicians, is a key milestone when validating a new diagnostic test to ensure the reliability of accuracy indices (i.e., sensitivity and specificity) (21).

In this study, we sought to assess the interobserver agreement in the interpretation of V/Q PET-CT for the diagnosis of acute PE, and to compare it to the interobserver agreement of CT pulmonary angiography (CTPA) interpretation.

## Methods and Materials

### Population

Patients included in the PECAN study were analyzed (20). The PECAN study is a prospective pilot study that assessed the independent and incremental value of V/Q PET-CT and CTPA for PE diagnosis. The design and main results of the study have been previously described (20). Briefly, 24 cancer patients with suspected acute PE underwent V/Q PET-CT and CTPA within 24 h. The eligible study population consisted of patients aged 18 years or older with a diagnosis of malignancy who were referred for CTPA or V/Q scan for suspected acute PE at the Peter MacCallum Cancer Center, Melbourne, Australia, between October 2014 and September 2017. All patients underwent both ^68^Ga V/Q PET-CT and CTPA within 24 h following referral for suspected PE. The study was approved by the institutional ethics committee and all patients signed written informed consent.

### Image Acquisition

The V/Q PET-CT scan was acquired on a Discovery 690 PET-CT scanner (GE Healthcare, WI, USA) (20). Ventilation images were acquired after inhalation of “Galligas” prepared using a Technegas generator (Cyclopharm, Sydney, Australia) by adding ~200 MBq of ^68^Ga rather than ^99m^Tc to the carbon crucible but otherwise following the Technegas production method. The patients were placed in a supine position and inhaled Galligas using the standard ventilation technique. Ventilation images were then acquired over two bed positions. Each bed position was acquired for 5 min. Without the patient moving, ~50 MBq of ^68^Ga-MAA was then injected Perfusion PET images were acquired with two bed positions. Each bed position was acquired for 3 min.

Chest CTPA protocol at Peter MacCallum institution entailed intravenous administration of 350 mg/mL of iodinated contrast at 3–5 mL per second with timing optimized for the pulmonary artery using bolus tracking and automatic triggering. Imaging was performed after a small suspended breath hold. Two CT machines were utilized in this study. The majority of the cases utilized Siemens AS+, with detector-row configuration of multidetector of 128 × 0.6. The second CT machine was a Siemens FORCE (two patients only), with a detector-row configuration of multidetector 2 × 192 × 0.6. With Siemens AS+, single energy configuration utilized CAREDOSE software with 100–140 kVp. With Siemens FORCE, dual energy configuration utilized CAREDOSE and CARE kV software with tube A at 80–100 kVp and tube B 150 kVp with Sn filter. Gantry rotation times were 0.5 s for AS + and 0.28–0.5 s for FORCE. Thin slices at 0.6 mm in axial soft tissue and lung algorithms with standard MPR reformats sent to PACS.

CTPA images were acquired on a Siemens SOMATOM Definition AS+ (Siemens Healthcare, Erlangen, Germany) from 2014 until June 2016 and a Siemens SOMATOM Force (Siemens Healthcare, Erlangen, Germany) from July 2016. Acquisition was performed during the pulmonary arterial enhancement phase following intravenous injection of contrast agent.

### Interpretation

In this present study, V/Q PET-CT and CTPA scans were retrospectively reassessed independently by four nuclear medicine physicians and four radiologists, respectively. Physicians had different levels of expertise in reading V/Q scintigraphy and CTPA. Nuclear medicine physicians had 35, 20, 13, and 1 year of experience, respectively. Radiologists had 13, 8, 8, and 6 years of experience, respectively. For each modality, readers were from two different institutions: two readers from the Peter MacCallum Cancer Center, Melbourne, Australia, and two readers from the Brest University Hospital, Brest, France. Interpretation was blinded to the initial interpretation and any clinical information or imaging test result. For each modality, results were reported on a binary fashion (i.e., “PE” or “no PE”). V/Q PET-CT scans were read as positive if there was at least one segmental or two subsegmental mismatched perfusion defects. Due to the absence of validated criteria for diagnosis of PE using V/Q PET-CT yet, we chose to apply the most commonly used criteria for V/Q SPECT/CT interpretation (10, 22, 23). CTPA scans were interpreted as positive if there was a constant intraluminal filling defect compatible with a PE. The location and size of clots and perfusion mismatched defects were also reported.

### Analysis

Interobserver agreement was assessed by a Cohen's kappa based on the calculi form K = Po-Pe/1-Pe, where Po is the relative observed agreement among physicians, and Pe is the hypothetical probability of chance agreement. We calculated the kappa coefficient for each pair of physicians within each imaging modality (i.e., 1–2, 1–3, 1–4, 2–3, 2–4, 3–4). We calculated the Fleiss' kappa coefficient for evaluating the level of agreement between multiple raters for nuclear medicine physicians and radiologists, respectively (Tables 1, 2). A kappa score above 0.8 is considered as almost perfect interobserver reliability, 0.6−0.8 is substantial interobserver reliability, 0.4−0.6 is moderate interobserver reliability, 0.2−0.4 is fair interobserver reliability, and below 0.2 is slight interobserver reliability (24).

**Table 1:** Kappa coefficients between each reader and for multiple readers for nuclear physicians.

**Nuclear physicians**	**Kappa coefficients (CI 95%)**
1&2	0.647 (0.013; 1.000)
1&3	1.000 (1.000; 1.000)
1&4	1.000 (1.000; 1.000)
2&3	0.647 (0.013; 1.000)
2&4	0.647 (0.013; 1.000)
3&4	1.000 (1.000; 1.000)
Multiple readers analysis (1&2&3&4)	0.789 (0.626; 0.952)

**Table 2:** Kappa coefficients between each reader and for multiple readers for radiologist.

**Radiologists**	**Kappa coefficients (CI 95%)**
1&2	0.333 (−0.187; 0.853)
1&3	0.600 (0.177; 1.023)
1&4	0.333 (−0.187; 0.853)
2&3	0.238 (−0.444; 0.920)
2&4	0.619 (0.114; 1.125)
3&4	0.238 (−0.444; 0.920)
Multiple readers analysis (1&2&3&4)	0.394 (0.231; 0.558)

## Results

All of the 24 patients from the PECAN study were analyzed. Patient's characteristics are described in Table 3.

**Table 3:** Patients' characteristics.

**Characteristics**	**No. (%)**
Age, mean ±*SD*, y.	55 ± 16
Female sex	10 (40)
Malignancy	
Metastatic	16 (67)
Surgery <3 Mo	5 (21)
Chemotherapy <3 Mo	16 (67)
Radiotherapy <3 Mo	11 (46)
Respiratory	
Lung malignancy	11 (46)
Prior lung surgery	4 (17)
Prior chest radiation therapy	9 (38)
Prior venous thromboembolism (PE and/or DVT)	7 (29)
Chronic respiratory insufficiency	3 (13)
COPD	6 (25)
Pre-test clinical probability	
Low	6 (25)
Intermediate	16 (67)
High	2 (8)

**Figure 1:**
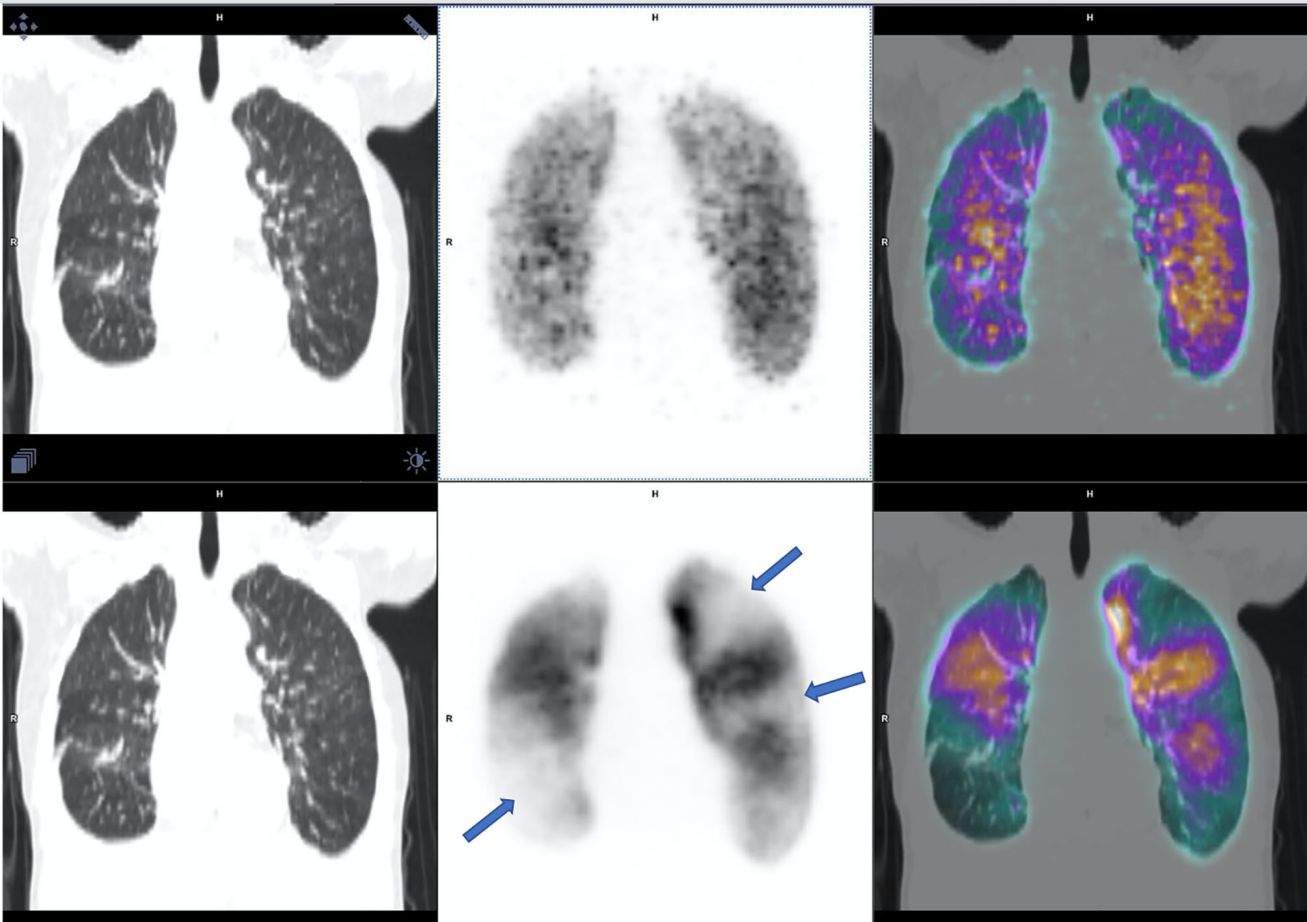
V/Q PET-CT, ventilation on the top and perfusion on the bottom, interpreted as positive by all of the four nuclear physicians with multiples mismatched perfusions defects (arrows).

**Figure 2:**
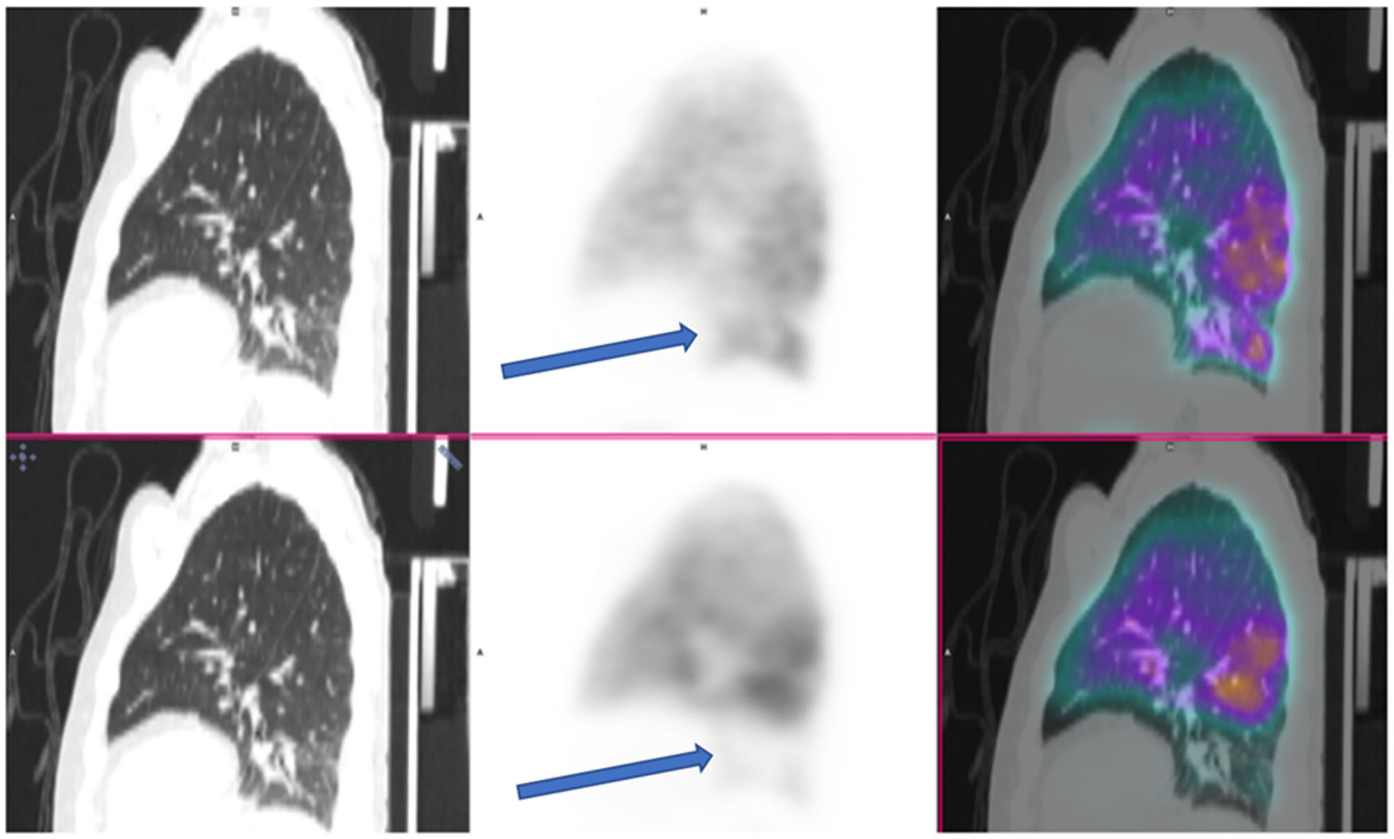
V/Q PET-CT, ventilation on the top and perfusion on the bottom, described as positive by one nuclear physician out of four with a mismatched perfusion defect on lower lobe (arrows), excluded by the three others because of a linear pattern of the defect and not wedge shape.

Median age was 58 years (range 21–79 years) and 10 patients were female. The clinical probabilities for PE as assessed by the revised Geneva score (25) were low, intermediate and high in six patients (25%), 16 patients (67%), and two patients (8%), respectively. Eleven (46%) patients had malignancy in the lungs, 4 (17%) had prior lung surgery and 9 (38%) had previously undergone chest radiation therapy. Out of the 24 V/Q PET-CT scans, the diagnostic conclusion of the four nuclear medicine physicians was concordantly negative in 22 (91%) patients and concordantly positive in 1 (4%) patient (Figure 1). The remaining scan was interpreted as positive by the reader with the lowest experience, and negative by the three others readers. This patient had a perfusion mismatched defect in the left lower lobe, in keeping with radiotherapy changes, which was blindly interpreted as positive for PE by one reader, while the three other readers ruled out the diagnosis of PE given the linear (and not wedge shape) pattern of the defect (Figure 2).

Out of the 24 CTPA scans, the diagnostic conclusion of the four radiologists was concordantly negative in 16 (65%) and concordantly positive in one (4%).

Out of the 7 (29%) remaining scans, PE was reported by only one of the four readers in four cases. The PE suspicion was described at a sub segmental level for three of them, and at a segmental level in one and notified as a possible chronic lesion. None of these four cases were reported as PE by the nuclear medicine physicians.

**Figure 3:**
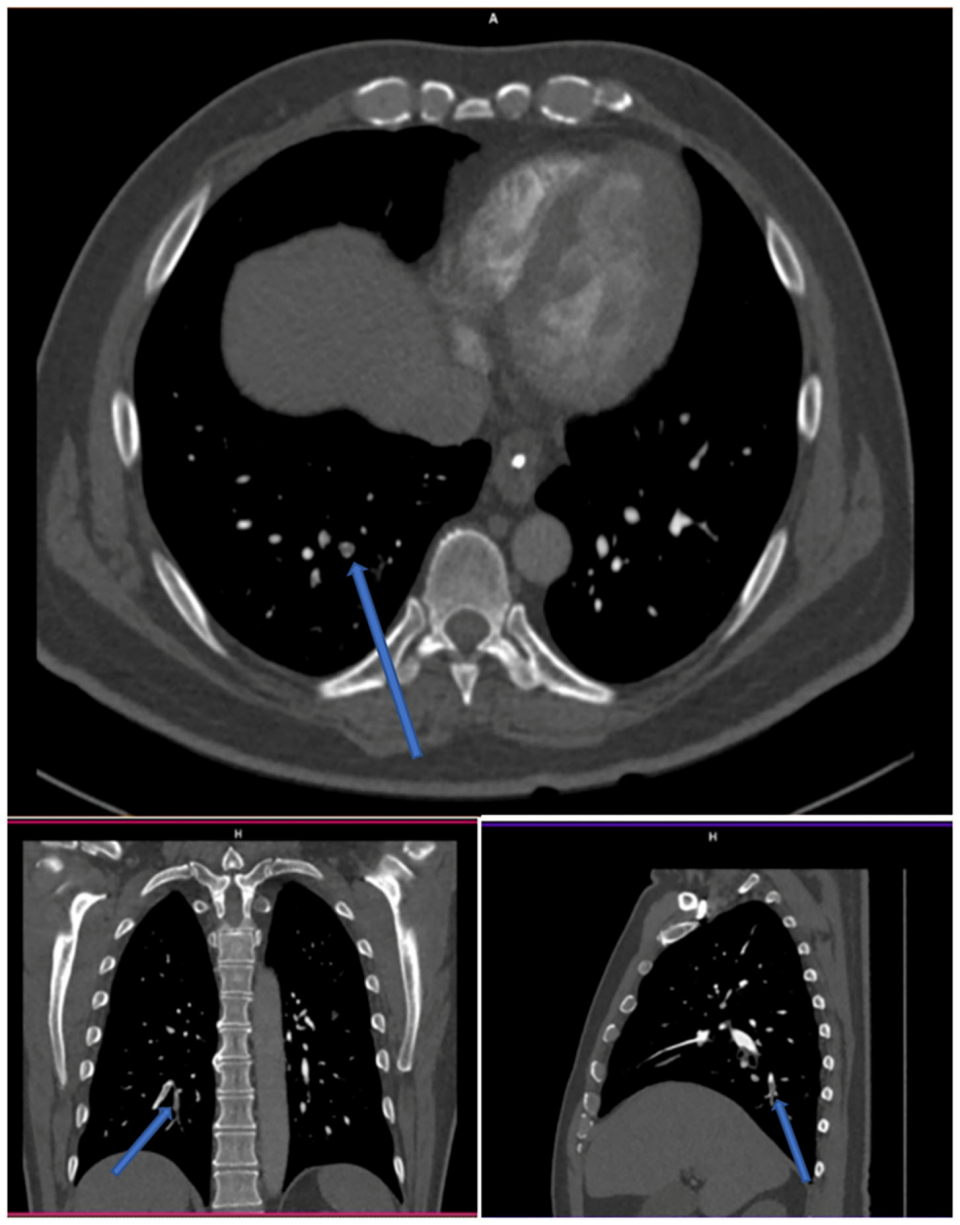
CTPA with endoluminal defect (arrows) reported by two radiologists out of four.

Two others cases were reported as positive for PE by the same two readers, who concomitantly described two subsegmental emboli in the same location, but one radiologist described two additional subsegmental clots (Figure 3, example of one discrepancy on CTPA scan). One of these two cases was the patient whose V/Q PET-CT scan was reported as positive by the four nuclear medicine physicians.

One case was reported as PE by three readers, concomitantly in the same subsegmental artery (left A5) for two radiologists but with a different location for the third physician who described a defect in subsegmental artery A6 and in segmental artery A9 on left lower lobe. None of nuclear medicine physicians reported a PE in this patient.

Out of the 7 CTPA with discordant conclusion between the four readers, 5 (71%) were due to clots reported on the subsegmental arteries.

Kappa coefficient was 0.79 between all nuclear physicians which is substantial, and 0.39 between all radiologists which is moderate.

## Discussion

In this study, the interobserver agreement in the interpretation of V/Q PET-CT for PE diagnosis was “substantial” with a mean kappa coefficient of 0.79. In contrast, the interobserver agreement in the interpretation of CTPA was fair, with a mean kappa coefficient of 0.39. This was mainly explained by discrepancies in identifying clots within sub-segmental arteries.

A first condition to be met before utilization of a new imaging test in clinical practice is that the interpretation is reproducible between different observers. Furthermore, in studies assessing the accuracy of a new imaging tool, the interpretation is often performed by readers with expertise in the field. A thorough knowledge of the disease and of imaging pitfalls may lead to an overestimate of the of the new test's performance. Accordingly, before conducting a formal diagnostic accuracy study, the interobserver reproducibility should be verified to ensure the relevance of future results and their generalizability in clinical practice (21).

In this study, the interobserver agreement in the interpretation of V/Q PET-CT for PE diagnosis was “substantial” with a mean kappa coefficient of 0.79. Only one out of 24 cases was discordant. As a comparison, in a cohort of 100 cases from Le Blanc et al. (26), the interobserver agreement in V/Q SPECT interpretation was also almost perfect, with kappa values ranging from 0.877 to 0.927. In our series, V/Q PET-CT scans were interpreted by four readers with varying experience, including a physician with only 1 year of training, showing a relative ease of learning and interpretation.

In contrast, the interobserver agreement in the interpretation of CTPA was lower, with a mean kappa coefficient of 0.39, mainly as a result of discrepancies in the subsegmental arteries (in five out of seven discordant cases). These results are consistent with previous interobserver agreement study of CTPA, which showed a low inter-observer variability between radiologists in the interpretations of subsegmental PE (27). In an interobserver agreement study involving five radiologists, and 290 CTPAs, the mean kappa value was 0.38 for emboli in the subsegmental vessels (k range 0.0–0.89) (28, 29). Furthermore, there is no clear consensus regarding management of patients with isolated subsegmental PE (30, 31). According to the recent ESC Guidelines for the diagnosis and management of acute pulmonary embolism (32), if the CTPA report suggests single subsegmental PE, the possibility of a false-positive finding should be considered. The findings should be discussed again with the radiologist or another reader to avoid misdiagnosis, and unnecessary, potentially harmful anticoagulation treatment. However, the results might suggest that CTPA is more sensitive than VQ PET-CT for small emboli. The prognostic significance of such discordant results will require larger studies.

Our work has some strength. To the best of our knowledge, this is the first study that assessed the interobserver reliability of V/Q PET-CT. For each modality, four physicians with different experience levels, from two different centers and countries, conducted the interpretation. Interpretation was blinded from each other, and from any clinical information and imaging test result. The inter-observer reliability was “substantial.” This positive result is encouraging prior to initiating further studies on the use of V/Q PET-CT for PE diagnosis.

Our study also has some limitations. Firstly, the cohort size was small. Furthermore, there was a relatively low prevalence of significant PE, with only one case of positive V/Q PET-CT, which is a limitation for the evaluation of interobserver agreement. Sample size was not determined a priori, since we used data from all patients included in the PECAN study. However, this is currently the largest series that assessed V/Q PET-CT in patients with suspected PE. Secondly, we were not able to compare the results to a ground truth. Indeed, as described in our prior study, both CTPA and V/Q PET-CT were used for patient management and some patients had insufficient follow up to provide a conclusive diagnosis (8). However, the aim of an interobserver agreement study is not to assess the accuracy of the test but to evaluate whether physicians would provide the same conclusion when reading a scan. Thirdly, we did not assess the intraobserver variability. Because of the small sample size with some typical and easily recognizable cases (e.g., the V/Q PET/CT scan with post radiation change), it was not possible to conduct a second unbiased interpretation. Fourth, the experience of nuclear medicine physicians and radiologists was not similar. Mean expertise of nuclear medicine physician was higher, and their level of expertise was more scattered.

In conclusion, in this study involving four nuclear medicine physicians with different expertise levels, we found a “substantial” interobserver agreement in the interpretation of V/Q PET-CT for PE diagnosis. This is an additional argument to support the use of V/Q PET-CT for PE diagnosis. Formal studies are now needed to assess the diagnostic accuracy of the test and, in particular whether a negative result carries a favorable prognosis without active intervention.

## Data Availability Statement

The original contributions presented in the study are included in the article/supplementary materials, further inquiries can be directed to the corresponding author/s.

## Ethics Statement

The studies involving human participants were reviewed and approved by Peter MacCallum Cancer Centre Ethics Committee 14/117. The patients/participants provided their written informed consent to participate in this study.

## Author Contributions

P-YLR and RL contributed to designing the study. P-YLR, MH, RH, and AI contributed to managing imaging procedures. P-YLR, P-YS, RH, MH, RL, BG, BW, AI, BD, and P-YLF contributed to reassessing all exams. MH, RH, GL, P-YLR, and RL contributed to analyzing the data. All authors contributed to writing the manuscript, read, and approved the final manuscript.

## Conflict of Interest

Unrelated to this study, MH reports grant funding from the Prostate Cancer Foundation,Movember, Prostate Cancer Foundation of Australia, Medical Research Future Fund (MRFF), Victorian Cancer Agency and the U.S. Department of Defence. He has received honorarium for educational lectures or travel from Janssen, Sanofi Genzyme and Ipsen. MH has received industry grant funding from Endocyte (now a Novartis company). The remaining authors declare that the research was conducted in the absence of any commercial or financial relationships that could be construed as a potential conflict of interest.
